# The Legal Status of Married Women

**Published:** 1914-05

**Authors:** Hortense Ward

**Affiliations:** Attorney at Law, Houston, Texas


### The Legal Status of Married Women.

BY HORTENSE WARD, ATTORNEY AT LAW, HOUSTON, TEXAS.

To fully appreciate the changes made by the last Legislature in the property rights of married women in Texas, a brief summary of her previous limitations and responsibilities is necessary.

Under the law prior to July 1, 1913, when a woman in Texas married, her husband had the sole management of all of her separate property and all her interest in the community. All her money, earnings, notes, securities and even her wearing apparel were completely under his control. He had the management of all of her property, without her consent and even against her will. He could draw out every cent of money that she might have in any bank, without her knowledge or over her protest. She could not exercise any control over any of her property without his permission, and then only at *his* agent, which permission and agency might be withdrawn at any time. He could lease or rent her property and collect and spend all revenue therefrom. He could dispose of all community property without her knowledge or consent, except the homestead. He could contract debts against the income of all her separate property and thereby prevent her or her children receiving any benefits therefrom. Her earnings were collectible by him only, and she could not collect or spend either her earnings, her separate funds or her income without his consent. While it was constantly reiterated that under the Texas laws married women retained all their property rights as to the title, still by giving the husband the sole management and control, and not holding him accountable, it resulted disastrously to any woman who happened to marry a man who was either dishonest or lacking in ability—and the woman could not raise her hand to save herself or her children from the effects of such dishonesty or inability.

Under the law known as the "Property Rights Bill," passed by the Thirty-third Legislature, all this is changed very materially. Now a married woman has the sole management, control and disposition of all her separate property, both real and personal, except that she can not sell or encumber her real property without the joinder of her husband, nor sell her stocks and bonds without his consent. But, in the event he refuses his consent, she may have her disabilities of coverture removed in an ex parte proceeding in the district court in the county of her residence, either in term time or vacation, and convey without his joinder. So that now we need not have cases like one shortly before the law was changed where a man compelled his wife to give him five hundred dollars to sign his name to transfer of a piece of land which had been left her by her father. She had three small children and was supporting the family. A married woman now can collect her own earnings, rents, interest on money, and dividends on stocks and bonds, and her husband not only can not collect them except as her agent, but these funds, while retaining their community character, are not subject to the debts of the husband, as they were under the old law.

The intention of the law was to put the woman in a position to control and handle her separate moneys, and the income from her separate property, and to hold it against any debts that the husband might contract. It does not, however, exempt from his control and debts the income of her separate property that might require any exercise of his skill or labor; for example, crops grown on the wife's separate lands are still under the husband's management and subject to sale by him.

A married woman, however, in gaining new liberties also assumed new responsibilities; she can no longer plead coverture to avoid her honest obligations; she must pay what she owes. If she agrees to do a thing, she must, or be answerable therefor. She can no longer make contracts and sign notes, and then change her mind and plead coverture. In other words, the law says she can no longer be a "welcher." And she must now abide by her word as she would have done without the aid of the law. Also, while a husband must furnish necessaries to his wife and her children after her marriage with him, he is not liable for her antenuptial debts nor is the community, except that part of it that is created by her earnings or the income from her separate property. The wife can not bind the separate property of the husband except for necessaries furnished after her marriage with him. All of the community is likewise liable for these; and that part of the community that is composed of her earnings or rents or revenues from her separate property is liable for all debts contracted by the wife.

The law as it is very favorable to married women; much more so than the laws of those States in which the separate property of each and all incomes therefrom and all earnings remain the separate property of either party, because under the community system the partnership in the result of combined efforts makes it much better for the wife, who is the homemaker, and at the same time it protects her in the income of her separate property, as it has always protected the man, by giving her the control of that part of the community.

There is nothing unfair in the law as amended, and it improves on investigation. Years of contrary thinking and centuries of prejudice make it difficult for many people, and especially many lawyers, to accept fully the changes made by the law. This is not strange to understand. Our laws are based on precedent, and, as a rule, the laws are decades behind progress, and the last van in the march is usually full of protesting attorneys; but they are not to blame for this, it's their educational training.

In all suits against a married woman she must still be joined by her husband pro forma. A married woman can not go on a bond as surety for another or be a joint maker of a note without being joined by her husband, but when she does so, joined by her husband, she is liable thereon. She, however, is liable on her own note or bond without his joinder, and it is only as joint maker or surety that she must secure his joinder to render her liable. The reason for this being that some of the lawmakers feared she might be overly pursuaded to be a joint maker or surety.

There is nothing in the law as amended which makes it obligatory for women to manage and control their own property, and in most cases, now as heretofore, the husband is managing and controlling both his wife's separate property and the income therefrom. In most cases this is by far the best arrangement, because, as a rule, he is better prepared and more able to do so. His sole object is to protect and take care of the wife and babies, and not only preserve to her all that is hers, but to add to it all that he can for her comfort and enjoyment. But in those sad and unfortunate cases where the wife needs protection against the man she has married, the Texas law now steps in and shields her as far as it can from the result of her error.

The law is not perfect; few laws are, and certainly after the stormy and turbulent journey it had, and the stabs made at it by the unthinking, the amendments tacked on by the unknowing, and the many trips back and forth, it is to be congratulated that it retained even a semblance of its former self. Few bills have had a more strenuous struggle for existence, and none that has passed since the adoption of the Constitution has so changed the status of the property rights of this State. There is only one other step that will more completely "emancipate" woman from the position of "chattel" that she formerly occupied, and that is the ballot—and it's coming.

